# Evaluation of educational interventions on eye health for dietetic and pharmacy professions: a pre-post study

**DOI:** 10.1186/s12909-021-02905-3

**Published:** 2021-09-07

**Authors:** Diana Tang, Helen Dinh, Hadi Almansour, George Burlutsky, Jocelyn Bussing, Bronwyn Eisenhauer, Bamini Gopinath, Victoria M Flood, Bandana Saini

**Affiliations:** 1 Centre for Vision Research, Department of Ophthalmology and Westmead Institute for Medical Research, University of Sydney, 176 Hawkesbury Road, New South Wales 2145 Sydney, Australia; 2 Macquarie University Hearing, Macquarie University, North Ryde, New South Wales Australia; 3 Sydney Pharmacy School, Faculty of Medicine and Health, The University of Sydney, Camperdown, New South Wales Australia; 4 Food and Nutrition Australia, Sydney, Australia; 5 Sydney School of Health Sciences, Faculty of Medicine and Health, The University of Sydney, Sydney, New South Wales Australia; 6 Western Sydney Local Health District, Westmead Hospital, Westmead, New South Wales Australia

**Keywords:** Age-related macular degeneration, Eye health, Pharmacist, Dietitian

## Abstract

**Background:**

We piloted an educational intervention that aimed to enhance awareness about nutrition-age-related macular degeneration (AMD) links among practising and student dietitians then expanded the scope of this intervention to include general eye health, which was delivered to pharmacy students.

**Methods:**

A pilot intervention was conducted in 2019 at the Dietitians Australia Conference (Gold Coast, Australia) where practising and student dietitians underwent a 2-hour small group educational workshop on nutrition and AMD links. Pre-post questionnaires were administered to participants, with voluntary completion of both questionnaires an indicator of consent to participate in the intervention. The primary intervention outcome was a change in AMD-related nutrition knowledge pre-post intervention. A larger intervention was then conducted at the University of Sydney (Sydney, Australia) where pharmacy students underwent a 4-hour educational module to improve general eye health knowledge, as well as student perceptions and attitudes towards a pharmacists’ role in low vision care. Similarly, pre-post questionnaires were administered, with voluntary completion of both questionnaires an indicator of consent to participate in the intervention. The primary intervention outcomes were changes in total knowledge, total perception and total attitude scores pre-post intervention.

**Results:**

(1) Among 10 accredited and 5 student dietitians, there was significant overall knowledge improvement (mean pre-post score: 7.07 ± 1.94 vs. 10.8 ± 1.01, *p* = 0.001) specifically around appropriate dietary advice, food sources of key AMD-related nutrients, and awareness of supplements. (2) Among 179 second-year pharmacy students enrolled in the ‘Pharmacy Practice’ Unit of Study (Bachelor of Pharmacy, University of Sydney), total eye health knowledge (6.25 ± 1.93 vs. 6.64 ± 2.0; *p* = 0.011) significantly improved, along with total perception scores (41.54 ± 5.26 vs. 42.45 ± 4.95; *p* = 0.004). Total attitude scores were not significantly different.

**Conclusions:**

The pilot intervention improved relevant nutrition-AMD knowledge among practising/student dietitians. The modified intervention for pharmacy students also significantly improved general eye health knowledge as well as students’ perception of a pharmacists’ role in low vision care.

**Supplementary Information:**

The online version contains supplementary material available at 10.1186/s12909-021-02905-3.

## Background

High quality eyecare involves a collaborative, multidisciplinary approach [1, 2]. This approach is recognised in the care of patients diagnosed with diabetic retinopathy where patients are referred to podiatrists, endocrinologists and nutritionists, in addition to eyecare practitioners and general medical practitioners [2, 3]. However, for other eye conditions, referral pathways are almost exclusively between eyecare practitioners and low vision rehabilitation services [2].

Support from additional health care professionals such as allied health care practitioners is warranted, particularly to address modifiable lifestyle risk factors for eye disease and injury[1]. One example of this is in the treatment of age-related macular degeneration (AMD), a leading cause of irreversible blindness [4]. Research literature and clinical practice guidelines[5] recommend smoking cessation and dietary improvements including regular consumption of dark green leafy vegetables [6], low glycaemic index (GI) foods [6–8], fish [4, 6, 9], as well as appropriate use of Age-Related Eye Disease Study (AREDS) nutritional supplements[10, 11] to reduce the risk of AMD development and progression. The original AREDS formulation consisted of 500 mg of vitamin C, 400 IU of vitamin E, 15 mg of beta carotene, 80 mg of zinc as zinc oxide and 2 mg of copper as cupric oxide, while the AREDS 2 formulation replaced beta carotene with 10 mg lutein and 2 mg zeaxanthin due to concerns regarding increased lung cancer risks associated with high dose beta carotene supplementation [10, 11]. Therefore, collaborative support from dietitians, as the experts in nutrition counselling, and pharmacists, as suppliers of nutritional supplements, could be valuable. Pharmacists in particular, also have a clientbase that extends beyond those purchasing AREDS supplements such as clients seeking other eyecare medicines e.g. lubricating eyedrops, and clients seeking non-eyecare medicines or services who may also present with low vision or blindness.

Despite the likelihood of engaging with visually impaired clients, research literature from Korea suggests that most pharmacists lack the skills to effectively communicate with these clients [12]. In this study, only 39 % used effective communication methods such as message reiteration and verifying listener comprehension, and 36 % used assistive technologies [12]. Further, of the 114 visually impaired consumer participants, 62 % reported a need to receive more detailed medicines information from healthcare professionals such as pharmacists, and 68 % indicated currently receiving no special counselling from their pharmacist [12].

As the pharmacy profession is evolving from ‘medication supply’ only roles to the provision of more involved health care services, it is imperative that pharmacists develop skills and knowledge to maintain continuity of care for people with visual impairment and for people with disability in general [13]. To address existing lacunae in pharmacist-provided patient-centred care for people with visual impairment, pre-registration level training needs to address such skills before imminent entry into the profession. However, to the best of our knowledge, there is a lack of published literature on professional educational training in this area for pharmacists.

Therefore, this study is novel and aims to evaluate the efficacy of a modified educational intervention to improve general eye health knowledge and perceptions and attitudes towards a pharmacists’ role in low vision care amongst pharmacy students. This aim will be achieved by: (1) testing the effectiveness of a pilot educational intervention to improve knowledge about nutrition-AMD links for practising and student dietitians and; (2) expanding on this intervention to include general eye health topics and education on clinical communication skills to deliver to pharmacy students at the University of Sydney.

## Methods

### Study overview

**Fig. 1:**
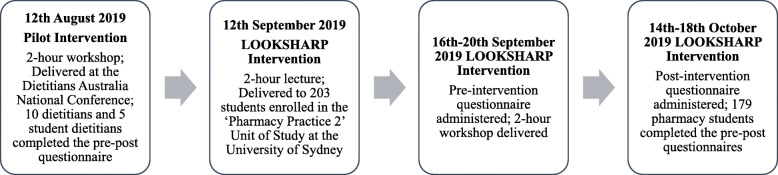
Timeline of the pilot and LOOKSHARP interventions

The pilot intervention was conducted on the 12th August 2019 at the Dietitians Australia National Conference, Gold Coast, Australia. This intervention consisted of a two-hour small group workshop on nutrition-AMD links. Delegates attending this workshop were provided a Participant Information Statement (PIS) about the intervention at the start of the workshop and informed that the voluntary completion of the anonymous pre-post questionnaires indicated consent to participate in the intervention. A feedback questionnaire was also included at the end of the post-intervention questionnaire. The pilot intervention was then modified for undergraduate pharmacy students by expanding the contents of the intervention to incorporate existing learning areas within the ‘Pharmacy Practice 2’ (PHAR2822) Unit of Study (UoS) curriculum. These learning areas included general eye health topics as well as education on clinical communication skills to prepare students with the skills to provide eye health advice to both normal sighted and vision-impaired clients. This intervention entitled ‘LOOKSHARP’, was delivered from the 12th September 2019 (Week 6, Semester 2) at the University of Sydney, Sydney Pharmacy School. LOOKSHARP consisted of a two-hour lecture on general eye health and AMD followed by a two-hour interactive small group workshop delivered the following week (Week 7, Semester 2). A research team member who was not involved in any related teaching or activities invited second-year students enrolled in ‘Pharmacy Practice 2’ to voluntarily participate in the intervention. As with the pilot intervention, students were provided a PIS at the start of the workshop and informed that voluntary completion the pre-post questionnaires indicated consent to participate in the intervention. Students who did not complete or return both questionnaires were excluded from the intervention. Questionnaires were administered immediately before (Week 7, Semester 2, 2019) and one-month after (Week 11, Semester 2, 2019) the workshop. A feedback questionnaire was also included at the end of the post-intervention questionnaire. Codes associated with each students’ questionnaire were only known by the administering team member and destroyed after post-intervention data collection in order for responses to remain anonymous. Figure 1 illustrates the timeline of the two interventions within this study. The design of these interventions were conducted in accordance with the Declaration of Helsinki and approved by the University of Sydney Human Ethics Committee (Reference: HREC2019/573 and HREC2019/575, respectively.)

The purpose of the pilot intervention was to determine if the educational content could significantly improve AMD-related nutrition knowledge among practising and emerging nutrition experts (i.e., dietitians). Significant improvement in this outcome was an indicator that the pilot intervention could be feasibly modified to improve awareness about relevant AMD-related nutrition links, such as appropriate use of nutritional supplements, among other allied health professionals such as pharmacy students, who are not nutrition experts but are suppliers of nutritional supplements.

### Pilot intervention

This intervention was developed by Accredited Practising Dietitians in collaboration with ophthalmologists and research experts in the field of AMD, and primarily consisted of: a presentation about current scientific evidence around nutrition and AMD, and three interactive activities. Activity 1 aimed to simulate the effects of AMD on vision and raise awareness around the implications of central vision impairment. Simulation glasses were ordered from Vision Australia, and attendees were provided different sized nutrition information panels to mimic an activity of daily living. Activity 2 assessed knowledge of food sources of lutein and zeaxanthin. Attendees were provided images of nine food items with specified portion sizes and asked to arrange in order of highest to lowest lutein and zeaxanthin content. Upon completion, facilitators presented the correct order according to the United States Department of Agriculture (USDA) National Nutrient Database [14]; discussed the relationship between lutein and zeaxanthin as well as factors that may influence their bioavailability. The final activity involved completing a case study to allow participants to apply their learnings to practice. Within smaller groups, attendees were asked to discuss one of three randomly allocated case studies including a dietary assessment, behaviour change strategies as well as developing a sample one-day meal plan. Case studies covered three different scenarios: (1) current smoker with early AMD; (2) healthy patient with family history of AMD; and (3) an older patient with neovascular AMD in one eye. Findings for each case study were reported back to the larger group.

To help participants retain the learnings from the workshop, off-the-shelf and independently developed resources were also provided to all attendees. These included publicly available resources from the Macular Disease Foundation Australia such as a ‘*Nutrients and Supplements’* brochure targeted at health professionals and a recipe booklet incorporating recommended foods for AMD management. In addition, researchers developed a one-week menu plan (Additional File 1) based on current Australian Dietary Guidelines and literature on nutrition and AMD, and an information sheet on lutein and zeaxanthin.

### LOOKSHARP intervention

This intervention was offered to Pharmacy students at the University of Sydney, Sydney Pharmacy School which offers a four-year program covering physiology, pharmaceutical science, health professional communication skills development and clinical knowledge for primary health care (including eye care) in the first two years followed by a focus on diseases across the body system, developing advanced clinical applications and clinical placements in the final two years.

LOOKSHARP was housed in the ‘Pharmacy Practice 2’ UoS, offered in the second half of Year 2, which included a focus on information delivery to patients about over-the-counter and complementary medicines [15]. This UoS follows ‘Pharmacy Practice 1’(PHAR1821) which broadly introduced students to patient communication skills, non-prescription medicines, clinical decision making and pharmaceutical care.

Table 1 describes the LOOKSHARP intervention. The education on AMD-nutrition links including workshop activities was modified from the pilot intervention. The overall structure of LOOKSHARP was based on Wilson and Lieberman’s three-level method [16] and associated learning objectives and outcomes were based on the Structure of the Observed Learning Outcome (SOLO) Taxonomy (Table 1) [17].

**Table 1:** Description of how the LOOKSHARP intervention and learning objectives were scaffolded based on the three-level method [16] and SOLO taxonomy [17]

Description of Wilson and Lieberman – three-level method [16]	Application of the three-level method to the LOOKSHARP intervention	SOLO Taxonomy [17]	Learning Objectives of the LOOKSHARP intervention
**Level I (Exposure)**: introduce pharmacy students to eye conditions such as AMD to allow students to achieve greater understanding and acceptance of people living with vision loss [16]	A two-hour lecture (scheduled in Week 6, Semester 2, 2019) on general eye health and AMD was delivered one week prior to the educational workshop by experts (dietitian and optometrist) and research team members. This allowed pharmacy students to have greater knowledge and formulate questions prior to the workshop.	**Unistructural**: students need to first grasp individual knowledge bits [17] **Multi-structural**: be able to tie and file these individual bits into meaningful concepts [17]	Increase awareness about the disability and functional limitations experienced by individuals with vision loss/vision impairment.
**Level II (Experience)**: allows opportunities for pharmacy students to actually experience daily obstacles experienced by people with vision loss [16]	A two-hour workshop (scheduled in Week 7, Semester 2, 2019) was facilitated by a registered pharmacist from the University of Sydney. The workshop included two activities that provided hands-on experience of a person with an eye condition. This included use of AMD simulation glasses, identifying foods that are key sources of important macular carotenoids (lutein and zeaxanthin) and eye drop demonstration role-plays to enhance the understanding of living with vision loss/ an eye condition. Details of these activities are covered in Additional File 2.	**Relational**: learn to relate concepts together [17].	Develop empathetic and appropriate communication skills to counsel patients about and supply (if relevant) key evidence-based products for AMD. Develop and apply a range of practical strategies to assist people with vision loss.
**Level III (Ownership)**: encourages pharmacy students to take responsibility to ensure that people with vision loss are treated equitably [16]	Case-study activities on eye conditions including AMD were included in the workshop to encourage students to reflect upon individual circumstances and professional roles to provide tailored advice and counselling (e.g. about nutritional supplements). A reflection activity was also included at the conclusion of the workshop. These activities encouraged students to reflect upon their roles in ensuring equitable health care for those with vision loss or other eye conditions. The UoS Coordinator also scheduled an assessment that required submission of a reflective essay on topics covered in workshops (including the LOOKSHARP workshop) in Week 13 (final semester week).	**Extended abstract**: apply learning to create new knowledge or reflect upon how the knowledge relates to reality [17]	Reflect upon pharmacist roles in extending care to people living with vision loss.

### Outcome measures

For the pilot intervention, knowledge change was measured using pre-post workshop questionnaires (Additional File 3). This consisted of six questions: one multiple choice clinical AMD question (Question 1); three multiple choice food knowledge questions (Questions 2–4); one yes/no awareness of AREDS supplements question (Question 5) and if the response to this was a ‘yes’, a free response comments box was provided to elaborate on supplement brand and AREDS formulation; and a final question to indicate profession (dietitian, student dietitian or other), and years practicing, if a dietitian (Question 6). Pre-post questionnaires were scored to assess overall knowledge change (correct = 1, incorrect = 0). For questions with multiple correct answer options, one point was allocated to each correct option selected. For Question 2, one additional score was awarded if appropriate ‘other’ dietary advice was provided, while for Question 5, one point was allocated if participants correctly mentioned or described AREDS supplements. Maximum possible scores for each question were: Question 1 = 1, Question 2 = 5, Question 3 = 3, Question 4 = 3, Question 5 = 1; with a maximum total score of 13.

The feedback form consisted of a 5-point Likert scale to assess overall satisfaction with the workshop; response options ranged from ‘Very dissatisfied’ to ‘Very satisfied’. A ‘yes/no’ question was also included to indicate if the participant would recommend the workshop to others, and a free response question was included to collect further detailed feedback and/or comments. Both questionnaires and feedback form were provided as one booklet to ensure pre-post questionnaire responses were matched to the same participant and a coversheet was attached to maintain anonymity by preventing researchers from seeing whether the survey had been completed at collection. Completion of both surveys indicated consent to participate in the intervention.

Similarly, outcomes of the LOOKSHARP intervention were assessed using pre-post questionnaires. The pre-workshop questionnaire comprised of four sections (Additional File 4):

- Sec. 1: Demographics

This section included seven ‘yes/no’ or a short answer questions regarding age, gender, country of birth/length of stay in Australia, employment, previous awareness about vision disorders and family history of vision impairment.

b) Sec. 2: Clinical Decision Making

This section included 12 forced-choice questions regarding supplements, dietary advice, preventative measures, symptoms, risk factors and facts about AMD. Questions were adapted from an existing questionnaire used to assess current practice of United Kingdom-based eye-care professionals in relation to dietary advice and lifestyle modifications for patients with or at risk of AMD [18]. Modifications were made based on a thorough literature review and the authors’ expertise. Responses to each item (1 = correct, 0 = incorrect) were added to calculate a maximum score of 12 (score ranged from 0 to 12).

iii) Sec. 3: Role of pharmacists in AMD/vision impairment/vision loss

This section included 12 items related to students’ perceptions about the role of pharmacists when interacting with people with vision impairment on a 5-point Likert scale. Questions were similarly adapted from an existing questionnaire [18] as described above. Response options ranged from ‘1 = Strongly disagree’ to ‘5 = Strongly agree’, with a combined total maximum score of 60 (score ranged from 12 to 60).

iv) Sec. 4: Perceptions about communication with individuals who have a disability

This section included 20 items from the validated ‘Interaction with Disabled Persons Scale’ (IDPS) [19] to evaluate how students communicate with people who have a disability on a 6-point Likert scale. Response options ranged from ‘-3 = Disagree very much’ to ‘3 = Agree very much’, with a combined total score that ranged from − 60 to 60. The IDPS was slightly modified to use ‘person first’ language (e.g. ‘disabled’ to ‘a person living with a disability’).

### Analysis

Data from both interventions were entered into SPSS® version 25 for analysis. For the pilot intervention, demographic data and participant feedback were analysed using descriptive statistics. For knowledge-based questions (Questions 1–5), the mean and range of scores were calculated. The scoring of Question 5 was determined based on their short answer responses i.e. correctly describing the AREDS formulation. The differences in scores were identified using Wilcoxon’s signed-rank test based on non-normal data distribution.

Demographic data from the LOOKSHARP intervention were similarly analysed using descriptive statistics. For Secs. 2, 3 and 4, the mean total knowledge, perceptions and attitude scores were calculated. The differences in scores were also identified using Wilcoxon’s signed-rank test due to non-normal data distribution. A reliability analysis using Cronbach’s alpha was undertaken for Secs. 2, 3 and 4 of the questionnaire.

## Results

### Pilot Intervention

A total of 15 delegates (10 dietitians and 5 student dietitians; 87 % female) attended the educational workshop on nutrition and AMD. Of the 10 qualified dietitians, experience ranged from new graduates to 30 years. All attendees agreed to participate in the pilot intervention (100 % response rate).

Table 2 describes the mean and range of pre-post questionnaire scores. All participants became aware that AMD affects central vision (Question 1) and could recommend appropriate dietary advice (Question 2), with some participants also listing correct ‘other’ advice. Improvements were also observed for lutein and zeaxanthin content in food (Question 3) and awareness of AREDS supplements (Question 5). The score changes along with overall knowledge improvement were statistically significant (*p*-value < 0.05). Knowledge of omega-3 content in fish was unchanged (Question 4).

**Table 2:** Pilot intervention: scores of knowledge about nutrition and AMD

Knowledge questions	Mean score ± SD		*p*-value
	**Pre-intervention Actual Score;** **(actual score range)**	**Post-intervention Actual Score; (actual score range)**	
1. Affected visual field due to AMD (score range: 0–1)	0.66 **±** 0.49; (0–1)	1.00 **±** 0.00; (1–1)	*0.025*
2. AMD-related dietary advice (score range 0–5)	2.73 **±** 1.22; (0–4)	4.20 **±** 0.41; (4–5)	*0.002*
3. Food sources of lutein and zeaxanthin (score range 0–3)	1.47 **±** 0.99; (0–3)	2.93 **±** 0.26; (2–3)	*0.002*
4. Omega-3 fatty acid content in fish (score range 0–3)	2.13 **±** 0.52; (1–3)	2.13 **±** 0.52; (1–3)	1.000
5. AMD supplement awareness (score range 0–1)	0.47 **±** 0.52; (0–1)	0.93 **±** 0.26; (0–1)	*0.008*
**Total knowledge score** **(score range 0–13)**	**7.07 ± 1.94; (3–10)**	**10.8 ± 1.01; (8–12)**	***0.001***

Overall feedback about the workshop was positive with participants specifically acknowledging the interactive nature of the intervention and the resources provided. Twelve out of 15 (80 %) participants were ‘satisfied’ or ‘very satisfied’ with the workshop, with the remaining three participants not completing the feedback form. Of the responders, 100 % recommended the workshop to others.

#### LOOKSHARP intervention

The LOOKSHARP intervention was delivered to 203 students enrolled in ‘Pharmacy Practice’ in Week 6–7, Semester 2, 2019. Ten tutorial groups ran throughout Week 7 with 179 students (88.2 % response rate) completing the pre-post questionnaires. Most participants were female (*n* = 120, 67 %); aged 18–34 (*n* = 170, 95 %); born in Australia (*n* = 99, 55.3 %) and; currently working in a pharmacy (*n* = 112, 62.6 %). More than 50 % (*n* = 102) of participants reported having a relative/friend with a vision impairment, however, more than three quarters (*n* = 152, 84.9 %) had not previously participated in any educational program about vision disorders.

The intervention significantly improved overall knowledge about AMD (*p* = 0.011), in particular knowledge about *Evidence-based supplements* (3 question items; *p* = 0.005) (Table 3). Overall perceptions of the roles of pharmacists for visually impaired patients also improved signficantly (*p* = 0.004), with key improvements in five areas: *Demonstrating the use of eye drops* (*p* = 0.028), *Use of assistive technology* (*p* = 0.028), *Facilitating self-management* (*p* = 0.050), *Providing specialised assistance* (*p* = 0.006) and *Identifying a person with vision impairment based on obvious factors* (p = 0.001) (Table 4). Although no difference was observed for the overall attitude scores (*p* = 0.578), there was significantly less discomfort when making contact with individuals with a disability (1 question item; *p* = 0.043) (Table 5). The Cronbach’s alpha values for the knowledge, perceptions and attitudes sections were 0.5, 0.7 and 0.7 respectively.

**Table 3:** Total score of pharmacy students’ knowledge about AMD (Sec. 2)

AMD knowledge themes (12 items)	Mean score ± SD		*p*-value
	**Pre-intervention Actual Score (theme percentage score)**	**Post-intervention Actual Score (theme percentage score)**	
Evidence-based supplements (3Qs, score range 0–3)	0.81 $\pm$ 0.84 (27 $\pm$ 28 %)	1.03 $\pm$ 0.88 (34 $\pm$ 29 %)	*0.005*
General dietary advice (3Qs, score range 0–3)	2.58 $\pm$ 0.66 (86 $\pm$ 22 %)	2.59 $\pm$ 0.69 (86 $\pm$ 23 %)	0.866
Risk factors and Preventative measures (2Qs, score range 0–2)	1.03 $\pm$ 0.78 (34 $\pm$ 26 %)	1.10 $\pm$ 0.73 (37 $\pm$ 24 %)	0.309
Pathophysiology and Symptoms (2Qs, score range 0–2)	0.80 $\pm$ 0.62 (27 $\pm$ 21 %)	0.90 $\pm$ 0.60 (30 $\pm$ 20 %)	0.059
Pharmacological treatments (2Qs, score range 0–2)	1.08 $\pm$ 0.63 (36 $\pm$ 21 %)	1.08 $\pm$ 0.81 (36 $\pm$ 27 %)	0.959
**Total knowledge score** **(score range 0 − 12)**	**6.25**$\pm$**1.93**	**6.64**$\pm$**2.0**	**0.011**

**Table 4:** Total score of pharmacy students’ perception of pharmacists in vision impairment (Sec. 3) classified into specific roles

Roles of pharmacists (12 items)	Mean Score on 1–5 scale ± SD 1 = Strongly Disagree 5 = Strongly Agree		*p*-value
	**Pre - intervention**	**Post - intervention**	
**Provision of information**			
Demonstrating the use of eye drops	4.61 ± 0.61	4.48 ± 0.65	*0.028*
Modify counselling	4.36 ± 0.88	4.46 ± 0.74	0.102
Use of assistive technology	4.10 ± 0.84	4.24 ± 0.73	*0.028*
Should use simple language^a^	3.67 ± 1.15	3.68 ± 1.17	0.802
Direct counselling to the carer^a^	3.59 ± 0.94	3.46 ± 1.01	0.195
**Monitoring**			
Screening	4.07 ± 0.90	4.16 ± 0.82	0.278
Facilitating self-management	3.95 ± 0.77	4.07 ± 0.83	*0.050*
**Management**			
Provide accessible interface points	4.50 ± 0.66	4.51 ± 0.64	0.918
Provide staff training	4.30 ± 0.88	4.32 ± 0.71	0.853
Should not provide specialised assistance^a^	4.13 ± 0.80	3.89 ± 1.00	*0.006*
**Vigilant**			
Identify a person with vision impairment based on obvious visual factors e.g. a guide dog^a^	3.30 ± 1.05	2.99 ± 1.10	*0.001*
Finding that people with vision impairment are difficult to deal with^a^	3.63 ± 0.99	3.52 ± 0.96	0.228
**Total perception score (score range 12–60)**	**41.54 ± 5.26**	**42.45 ± 4.95**	***0.004***

^a^Negatively phrased items were reversed-scored before analysis, 1 = Strongly agree to 5 = Strongly disagree

**Table 5:** Total score of pharmacy students’ attitude towards communicating with individuals who have a disability (Sec. 4) classified into specific themes

Attitudes of pharmacy students (20 items)	Mean Score on -3–3 scale ± SD -3 = Disagree Very Much 3 = Agree Very Much		*p*-value
	**Pre -** **intervention**	**Post - intervention**	
**Social Discomfort**			
Uncomfortable^a^	0.84 ± 1.76	0.86 ± 1.73	0.916
Staring^a^	1.44 ± 1.58	1.30 ± 1.58	0.536
Unsure how to behave^a^	0.42 ± 1.75	0.66 ± 1.71	0.184
Brief contact^a^	1.81 ± 1.38	1.53 ± 1.61	*0.043*
Overwhelmed^a^	1.08 ± 1.69	0.95 ± 1.75	0.834
**Fear**			
Dread of having a disability^a^	-0.57 ± 1.82	-0.76 ± 1.81	0.593
Grateful for not having a disability^a^	-2.43 ± 1.08	-2.25 ± 1.20	0.307
Afraid to look straight in the face^a^	1.75 ± 1.48	1.54 ± 1.61	0.368
**Empathy**			
No pity	-0.66 ± 1.80	-0.32 ± 1.81	0.075
Notice the person and not the disability	1.53 ± 1.51	1.47 ± 1.51	0.964
Act normal and ignore the disability	0.97 ± 1.77	0.93 ± 1.87	0.826
Discuss about the disability	1.05 ± 1.59	1.04 ± 1.64	0.775
**Perceived level of information**			
Ignorant^a^	1.04 ± 1.87	0.88 ± 1.88	0.714
Aware of problems	1.15 ± 1.46	1.21 ± 1.52	0.833
**Vulnerable**			
Hurts to see a person with a disability	2.10 ± 1.18	1.89 ± 1.29	0.061
Frustrated when feeling useless	2.03 ± 1.16	1.83 ± 1.33	0.072
Reminder of one’s own vulnerability	0.34 ± 1.94	0.45 ± 1.87	0.671
Wonder what it is like to have a disability	1.32 ± 1.64	1.30 ± 1.64	0.990
**Enriched**			
Rewarding to help	2.43 ± 0.90	2.29 ± 1.67	0.248
Admire ability to cope	2.09 ± 1.12	2.09 ± 1.15	0.942
**Total attitude score (score range − 60–60)**	**19.18 ± 10.19**	**18.57 ± 11.20**	**0.578**

^a^Negatively phrased items were reversed-scored before analysis, -3 = Agree very much to 3 = Disagree very much

## Discussion

This is the first study to pilot an educational intervention on nutrition-AMD links for practising and student dietitians, which was then expanded in scope to educate pharmacy students about general eye health. These interventions were shown to be efficacious in both groups of participants regarding improvements in relevant AMD and eye health knowledge.

The pilot intervention significantly increased awareness about appropriate dietary advice for people with AMD among participating dietitians. In addition to improving knowledge about important food groups for AMD, participants’ awareness of food sources of lutein and zeaxanthin and the availability of supplements for optimal macular health also significantly improved. However, participants appeared to have only a moderate understanding of omega-3 fatty acid content of different fish, and this did not change by the end of the workshop. This is likely explained by the limited education on omega-3 fatty acids, which was only broadly covered in the overview presentation at the start of the workshop due to time constraints. On the other hand, the education on lutein and zeaxanthin was covered more extensively and included a specific smaller group activity followed by a wider group post-activity discussion. Moreover, education on AREDS supplements was extensively convered in the intervention, featuring in the overview presentation and case study activity to encourage participants to apply their learnings to real-life patient scenarios. Such interactive group activities and discussions align with the notion of authentic learning experiences that tend to lead to deeper-rooted learning [20].

The LOOKSHARP intervention for pharmacy students also significantly improved overall knowledge about AMD, specifically knowledge about evidence-based supplements. Non-significant improvements were evident for knowledge items focussed on risk factors and pathophysiology of AMD and general dietary advice. Improvement in particular items may be due to students’ prioritisation of potential examination topics according to the UoS outline [15, 21] or personal relevance as 62.6 % of students work in a pharmacy where knowledge about eye-health can be immediately applied. Notably, low-level improvement in other knowledge items may be due to high baseline knowledge, as observed in the ‘General dietary advice’ section where mean baseline score was 2.58 $\pm$ 0.66 out of 3.

LOOKSHARP also demonstrated significant improvement in the overall perception about pharmacists’ roles in managing people with vision impairment. This included stronger agreement around utilising assistive technology when counselling patients, suggesting that students acknowledged the benefit of such tools for effective communication. A meaningful shift in responses toward the neutral/disagreement end of the scale for the perception that ‘people with visual aids do not require assistance as they obtain it elsewhere’ demonstrates that the intervention contributed to more open-minded perceptions and disassociated common stereotypes about people with vision impairments. Interestingly, the score for agreement with pharmacist’s role around demonstrating eye drops decreased significantly post-intervention. This change in perception was possibly due to the hands-on experience with self-administering/peer feedback on eye drop technique. Also, after observing the facilitating tutor/ registered pharmacist’s demonstration then having to learn how to use and counsel others about eye drops reflected how participants may have overestimated their skills prior to the intervention.

Despite improvements in overall knowledge and perception about vision impairment in the pharmacy students, no significant difference was observed in attitude scores. This may be due to the use of the 1997 IDPS, which may be less sensitive to detect change in current students who are well exposed to changes in societal awareness about equity, accessibility and social responsibility [19]. Moreover, the intervention itself may have lacked the intensity to enact a change in attitudes as a US study using the IDPS on psychology undergraduate students reported comparable baseline attitude scores however achieved a positive change in attitude scores following ten-hours of service-learning that involved direct contact with people with a disability [19, 22]. Direct contact with people with a disability has also been shown to foster non-prejudicial attitudes as it allows a more personal insight to the struggles of people living with a disability and thus, can potentially impact on student attitudes [23, 24]. However, we propose that both intensive training and exposure to people with myriad vision disorders may be more effective, as exposure alone may not shift strongly entrenched attitudes. For example, the results from another educational intervention conducted with a similar pharmacy student cohort which utilised simulated patients with lived experience of suicide failed to change attitudes about suicide in pharmacy students, although it wrought positive improvements in confidence about counselling someone contemplating suicide [25].

Overall, the relevant knowledge gains among practising and student dietitians in the pilot intervention was successfully adapted to achieve similar outcomes among pharmacy students. However, the authors acknowledge several study limitations. Firstly, the pre-post study design lacks a control group and is known to be associated with response shift bias[26] despite literature suggesting that many educational intervention studies utilise the pre-post test method [27]. Future studies could consider retrospective pre-test to overcome this bias [26]. Secondly, both interventions included non-validated questions as they were custom constructed based on the combined expertise of the authors. Thirdly, the effects of the interventions on practice were not assessed as part of this study, and further follow up is needed to assess impacts on the clinical practice of dietitians and pharmacists. Finally, the respective interventions also had their own limitations. The pilot intervention had a small sample size and therefore, results may not be representative of the broader population of practising and student dietitians in Australia. Further, as different workshops were run concurrently at the Dietitians Australia conference, delegates choosing the nutrition and AMD workshop may have had a personal interest in the topic and there could have been resulting selection bias. For the LOOKSHARP intervention, data analysis of Sec. 2 (*Clinical decision making*) indicated a 0.5 Cronbach’s alpha value, which is below the arbitrary recommendation of 0.7 in reliability analysis [28].

Despite these limitations, the knowledge gains from the LOOKSHARP intervention has led to the ongoing inclusion of this module in the ‘Pharmacy Practice’ UoS at the University of Sydney, Australia.

## Conclusions

Educational interventions can enhance nutrition-AMD knowledge in practising and student dietitians, as well as improve relevant eye health knowledge and perceptions about a pharmacist’s role in low vision care in pharmacy students. The LOOKSHARP intervention has now been incorporated into the ‘Pharmacy Practice’ UoS curricula at the University of Sydney. Future research directions include the potential expansion of this educational intervention to other relevant healthcare professions such as general practice.

## Supplementary Information

**Additional file 1.**

**Additional file 2.**

**Additional file 3.**

**Additional file 4.**

## Data Availability

The datasets used during the current study are available from the corresponding author on reasonable request.

## Abbreviations

AMD: Age-related Macular Degeneration.
AREDS: Age Related Eye Disease Study.
IDPS: Interaction with Disabled Persons Scale.
PIS: Participant Information Statement.
SOLO: Structure of the Observed Learning Outcome.
UoS: Unit of Study.
USDA: United States Department of Agriculture.
