# Supplementary material for: Evaluation of educational interventions on eye health for dietetic and pharmacy professions: a pre-post study
Source: BMC Med Educ. 2021 Sep 7;21:478. doi: 10.1186/s12909-021-02905-3 (PMC8424804; doi:10.1186/s12909-021-02905-3)
Supplement: Supplementary file 1 — Additional file 1. [file 12909_2021_2905_MOESM1_ESM.docx]

| **Eye Health Meal Plan for Older People** | | | | | | | |
| --- | --- | --- | --- | --- | --- | --- | --- |
|  | **Meat-Free Monday** | **Tuesday** | **Wednesday** | **Thursday** | **Meat-Free Friday** | **Saturday** | **Sunday** |
| ***Breakfast*** | **Fruit Toast**  2 regular slices of fruit loaf  + 1 glass milk  + 1 medium orange | **Porridge with Chia**  1/2 cup dried rolled oats  + 0.5Tb chia seeds +  250ml milk  + mixed berries;  cinnamon to taste | **Peanut Butter on Toast**  2 slices of low GI bread (toasted) with 1/2 Tb peanut butter  1 glass milk  1 medium banana | **Baked Beans on Toast**  1 cup salt-reduced baked-beans on low GI toast  1 glass milk | **Porridge with Chia**  1/2 cup dried rolled oats  + 0.5Tb chia seeds + 250ml milk  + seasonal fruit;  cinnamon to taste | **Scrambled Eggs on Sourdough**  2 eggs +1 Tb milk scrambled. Serve on 1 slice sourdough toast + ¼ avocado. Top with flat-leaf parsley.  1 glass milk | **Cereal & fruit**  ¾ cup wholegrain flaky cereal sprinkled with pumpkin seeds + 250ml milk  1 medium orange |
| ***Lunch*** | **Corn and Veggie Fritters**  See recipe at: *https://www.australianeggs.org.au/recipes-and-cooking/* | **Tuna and Cucumber Sandwich**  2 slices low GI bread spread with mayonnaise, 1 small can tuna, cucumber slices & ½ cup baby spinach | **Feta and Spinach**  **Omelette**  See recipe at:  *https://www.australianeggs.org.au/recipes-and-cooking/* | **Red Lentil, Pumpkin and Tomato Soup (1 serve)**  Refer to the Macula Menu Recipe Booklet | **Salad & Cheese Sandwich**  2 slices low GI bread with 2 tsp olive oil spread + 1 slice cheese  + 1 salad vegetables  (e.g. cos lettuce, cucumber, tomato, red onion) | **Broccoli and Pea Soup**  Refer to the Macula Menu Recipe Booklet | **Roast Dinner**  100g lean pork + 1 medium baked potato + 1.5 cups baked vegetables (pumpkin, capsicum, zucchini) + 2 tsp olive oil (for cooking) |
| ***Dinner*** | **Tofu and Hokkien Noodle Stir-fry**  100g diced firm tofu  + 1.5 cup veg (capsicum, beans, carrot) + 1 cup Hokkien noodles + 2 tsp sesame oil + 2 Tb soy sauce. Top with sesame seeds | **Grilled Chicken & Vegetables**  100g lean chicken with 1 medium corn cob, 1.5 cups baked vegetables and 2 tsp olive oil (for cooking) | **Spaghetti Bolognese**  1/2 cup cooked lean mince with 1 cup cooked spaghetti, tomato, 30g grated cheese and 2 tsp olive oil (for cooking)  + 2 cups side salad | **Lamb Chops & Vegetables**  1 large trim lamb chop + 3/4 cup sweet potato +  1/2 cup steamed broccoli, ½ cup peas  + 2 tsp olive oil (for cooking) | **Red Lentil, Pumpkin and Tomato Soup (1 serve)**  (leftovers) | **Salmon, Rice & Vegetables**  100g salmon + 1/2 cup cooked basmati or doongara rice + 1.5 cups beans, peas & carrots | **Sardines on Toast**  5 tinned sardines (55g)  2 slices low-GI toast  Avocado, sliced tomato, flat-leaf parsley  Lemon squeeze |
| ***Dessert/***  ***Supper*** | 1 tub low fat custard | 1 cup canned peaches +  2 scoops ice-cream | 1 cup fruit salad | 1 tub low-fat yogurt  1 cup sliced strawberries | **Smoothie**  1 cup milk + 2 Tb plain yogurt + 1 small banana, 1 cup baby spinach, ½ cup frozen berries | 1 cup blueberries +  1 tub low fat custard | 1 cup fruit salad +  2 scoops ice-cream |
| ***Snacks*** | Handful mixed nuts and seeds (30g)  **Smoothie:** 1 cup milk + 2 Tb plain yogurt  + 1 small banana, 1 cup baby spinach, ½ cup frozen berries | Leftover Corn and Veggie Fritters  4 vita-wheat crackers with 2 slices cheese | 1 tub low fat yoghurt | 1 medium orange  Handful pistachio nuts (30g) | English muffin + 0.5Tb peanut butter | 1 cup diced rockmelon  1 tub low-fat yogurt | 1 tub low fat yoghurt  10 olives |

Additional File 1: Eye Health Meal Plan for Older People

Prepared by Accredited Practising Dietitians at Food and Nutrition Australia in collaboration with Professor Vicki Flood and Diana Tang. We acknowledge the support of Australian Eggs.2019

This meal plan meets the Australian Guide to Healthy Eating recommended average daily serves for adults 70 years and over, emphasising green leafy vegetables, citrus fruits, legumes, lentils, nuts and seeds. The plan provides:

- An average daily intake of lutein and zeaxanthin >6mg per day
- 2.5 fish meals a week
- Low GI carbohydrates throughout the week

For tailored advice please speak to an Accredited Practising Dietitian.
