# Supplementary material for: Evaluation of educational interventions on eye health for dietetic and pharmacy professions: a pre-post study
Source: BMC Med Educ. 2021 Sep 7;21:478. doi: 10.1186/s12909-021-02905-3 (PMC8424804; doi:10.1186/s12909-021-02905-3)
Supplement: Supplementary file 2 — Additional file 2. [file 12909_2021_2905_MOESM2_ESM.docx]

Additional File 2: Content of the LOOKSHARP workshop

| **Activity** | **LOOKSHARP Study details** |
| --- | --- |
| Pre-workshop questionnaire | Participants who consented to the study had 15 minutes at the beginning of the workshop to complete the questionnaire and these were collected by a research team member. All students were encouraged to complete the questionnaire and if they chose not to participate, to keep the questionnaire responses as a self-assessment activity. |
| Simulation glasses  (Experience Level)^11^ | Each students was given a pair of macular degeneration simulation glasses purchased from Vision Australia (<https://www.visionaustralia.org/>) and a dispensed label for an eye drop product*. Students had to wear the glasses and read the instruction on the label and reflect on the activity through a group discussion. |
| Lutein food source activity  (Experience)^11^ | Students were given images of a mixture of food items with specified portion sizes and asked to rank the food items in order of the highest to least amount of lutein according to the USDA National Nutrient Database for Standard Reference (<https://www.ars.usda.gov/>). |
| Case studies about age-related macular degeneration  (Ownership)^11^ | In small groups (5-6 students), students had to go through 3 cases related to macular degeneration. Each group was allocated to one of the cases about a patient with:   - early AMD and smoking history - family history of AMD but otherwise healthy - late AMD in one eye with the other eye unaffected   Students discussed appropriate recommendations for their allocated case particularly use AREDS supplements. |
| Case studies about general eye health  (Ownership)^11^ | Students were split up into pairs and received a case study about bacterial conjunctivitis. Students were required to do role-plays and peer-assess their paired peer’s skills in either clinical communication or skills in demonstrating eye-drop technique.* |
| Reflection activity  (Ownership)^11^ | Students were given a reflection card to reflect on their experience after attending the workshop. Students were asked to reflect on what they have learnt about people living with vision disorders and to consider and comment on how pharmacists could improve services for people with eye problems and vision disorders. |

*other common presentations related to eye health were used in this activity, given this workshop was the only curricular space allocated to eye health topics in this UoS
