# Supplementary material for: Evaluation of educational interventions on eye health for dietetic and pharmacy professions: a pre-post study
Source: BMC Med Educ. 2021 Sep 7;21:478. doi: 10.1186/s12909-021-02905-3 (PMC8424804; doi:10.1186/s12909-021-02905-3)
Supplement: Supplementary file 3 — Additional file 3. [file 12909_2021_2905_MOESM3_ESM.docx]

Additional File 3: Dietitians’ Workshop pre-post questionnaire

| **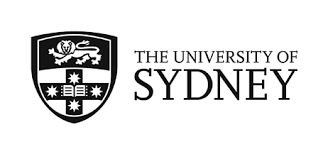** | **Nutrition and Age-Related Macular Degeneration:**  **Pre-workshop Survey** |
| --- | --- |
| This is a survey to help evaluate the effectiveness of the Nutrition and Age-related Macular Degeneration (AMD) workshop. Your responses may help to improve components of the workshop for future use. This survey will take about 5 minutes to complete. | |

1) AMD typically affects which area of vision? Tick one.

|  | Central vision |
| --- | --- |
|  | Peripheral vision |
|  | Not sure |

2) What dietary advice is generally recommended to reduce the risk of AMD development in a patient with a family history of AMD? Tick all that apply.

|  | Choose low GI foods |
| --- | --- |
|  | Eat dark green leafy vegetables regularly |
|  | Eat oily fish at least twice per week |
|  | Include a handful of nuts per week |
|  | Include yoghurt regularly |
|  | Other, please specify: |
|  | Not sure |

3) Which of the following are sources of the macular carotenoids (lutein/ zeaxanthin)? Tick all that apply.

|  | Carrots |
| --- | --- |
|  | Eggs |
|  | Olive Oil |
|  | Pistachios |
|  | Spinach |
|  | Not sure |

4) Choose from the following fish types, three (3) with the highest omega-3 fatty acid content (EPA and DHA) per equal serve?

|  | Barramundi |
| --- | --- |
|  | Canned Tuna, drained |
|  | Rainbow Trout |
|  | Salmon |
|  | Sardines |
|  | Snapper |

5) Are you aware of any nutrition supplements specific to AMD?

|  | Yes |
| --- | --- |
|  | 1. Please specify what nutrients are included in supplements specific to AMD: |
|  | 1. Please specify the names/brands of nutrition supplements specific to AMD: |
|  | Not sure |

6) Please indicate your profession:

|  | Dietitian, years practising: |
| --- | --- |
|  | Dietetic student |
|  | Other, specify: |

| **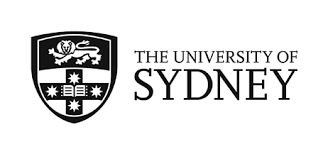** | **Nutrition and Age-Related Macular Degeneration:**  **Post-workshop Survey** |
| --- | --- |
| This is a survey to help evaluate the effectiveness of the Nutrition and Age-related Macular Degeneration (AMD) workshop. Your responses may help to improve components of the workshop for future use. This survey will take about 5 minutes to complete. | |

1) AMD typically affects which area of vision? Tick one.

|  | Central vision |
| --- | --- |
|  | Peripheral vision |
|  | Not sure |

2) What dietary advice is generally recommended to reduce the risk of AMD development in a patient with a family history of AMD? Tick all that apply.

|  | Choose low GI foods |
| --- | --- |
|  | Eat dark green leafy vegetables regularly |
|  | Eat oily fish at least twice per week |
|  | Include a handful of nuts per week |
|  | Include yoghurt regularly |
|  | Other, please specify: |
|  | Not sure |

3) Which of the following are sources of the macular carotenoids (lutein/ zeaxanthin)? Tick all that apply.

|  | Carrots |
| --- | --- |
|  | Eggs |
|  | Olive Oil |
|  | Pistachios |
|  | Spinach |
|  | Not sure |

4) Choose from the following fish types, three (3) with the highest omega-3 fatty acid content (EPA and DHA) per equal serve?

|  | Barramundi |
| --- | --- |
|  | Canned Tuna, drained |
|  | Rainbow Trout |
|  | Salmon |
|  | Sardines |
|  | Snapper |

5) Are you aware of any nutrition supplements specific to AMD?

|  | Yes |
| --- | --- |
|  | 1. Please specify what nutrients are included in supplements specific to AMD: |
|  | 1. Please specify the names/brands of nutrition supplements specific to AMD: |
|  | Not sure |

6) Please indicate your profession:

|  | Dietitian, years practising: |
| --- | --- |
|  | Dietetic student |
|  | Other, specify: |

**Workshop Evaluation and Feedback**

1) Rate your satisfaction with the workshop

| Very dissatisfied | Dissatisfied | Neither | Satisfied | Very satisfied |
| --- | --- | --- | --- | --- |

2) Would you recommend this workshop to others?

|  | Yes |
| --- | --- |
|  | No |

3) Do you have any suggestions to improve the workshop or any other comments?

|  |
| --- |
|  |
|  |
|  |
|  |
