# Supplementary material for: Evaluation of educational interventions on eye health for dietetic and pharmacy professions: a pre-post study
Source: BMC Med Educ. 2021 Sep 7;21:478. doi: 10.1186/s12909-021-02905-3 (PMC8424804; doi:10.1186/s12909-021-02905-3)
Supplement: Supplementary file 4 — Additional file 4. [file 12909_2021_2905_MOESM4_ESM.docx]

Additional File 4: LOOKSHARP study sample pre-questionnaire


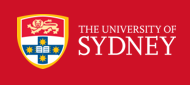


# *Role of pharmacists*

***in caring for people with***

***age-related macular degeneration***

***or other forms of vision impairment***

***or vision loss –***

***QUESTIONNAIRE (PRE)***

## **Student Unique Identification Number**

#### **Purpose and Significance**

This questionnaire is designed to assess second year pharmacy students’ awareness about their role in providing care for people with vision disorders, particularly with a focus on age-related macular degeneration (AMD). The questionnaire will be administered to second year pharmacy students at the University of Sydney before and after they complete relevant learning activities within the Pharmacy Practice 2 (PHAR 2822) Unit of Study. The results of this study will help us understand whether a specifically designed suite of educational resources and teaching delivery help enhance not only clinical awareness of AMD, but skills in communicating and helping people with low vision in the community pharmacy setting.

*As your feedback is important, your participation will be highly appreciated. We assure you that your responses will be completely confidential. The demographic information section in the questionnaire is asked to help us determine the overall characteristics of respondents and see if different people respond in a varied manner. Your unique identification number on the cover page is used only as a means of mapping responses before and after you undergo the above activities in PHAR 2822 and will not be used to identify any student. Course coordinators and subject markers will not have any access to your surveys.*

| **SECTION 1: DEMOGRAPHICS** |
| --- |

*PLEASE COMPLETE THE FOLLOWING SECTION BY PROVIDING ANSWERS WHERE INDICATED OR BY CIRCLING* *THE MOST APPROPRIATE CHOICE.*

| 1. **Age (in years):** | ................................... | | |
| --- | --- | --- | --- |
| 1. **Gender:** | Female | Male | Other |
| 1. **Were you born in Australia?** | Yes | No |  |
| 1. ***If Not*, have you been living in Australia for more than five years?** | Yes | No |  |
| 1. **Are you currently working in a pharmacy?** | Yes | No |  |
| 1. **Have you previously participated in a subject or program where you learnt about vision disorders?** | Yes | No |  |
| 1. **Do you have any relatives or friends who live with vision impairment?** | Yes | No |  |

| **SECTION 2: CLINICAL DECISION MAKING** |
| --- |

*THE FOLLOWING SECTION CONTAINS A LIST OF 12 STATEMENTS.*

*FOR EACH STATEMENT PLEASE SELECT THE MOST APPROPRIATE CHOICE BY PLACING ‘X’ IN THE BOX TO THE LEFT OF THE CORRECT CHOICE/S.*

| **1** | **Mr Smith is a 55-year-old patient with no evidence of age-related macular degeneration. His father had the condition and his older brother has also recently been diagnosed with AMD. Today he comes into the pharmacy and asks you for some nutritional supplements he has heard help prevent AMD**.  **WHAT SUPPLEMENTS, IF ANY, WOULD YOU SUPPLY HIM WITH?**   - Supplements containing Vitamins A, C, E and zinc e.g. Blackmores Macu-Vision - Supplements containing macular carotenoids (lutein and zeaxanthin) e.g. Lutein-Vision - Supplements containing Vitamin C, E, zinc, copper, lutein and zeaxanthin e.g. Macutec Essentials, Macu-Vision Plus - Supplements containing omega 3 fatty acids e.g. Lutein-Vision Advanced - None of the supplements listed above are appropriate for a patient at this stage |
| --- | --- |
| **2** | Mrs Bratislova is a 65-year-old regular patient of your pharmacy with advanced AMD in one eye and early AMD in the other. She presents one day and requests you to supply some nutritional supplements known to help People with AMDs?  **WHAT SUPPLEMENTS, IF ANY, WOULD YOU SUPPLY HER WITH?**   - Supplements containing Vitamins A, C, E and zinc e.g. Blackmores Macu-Vision - Supplements containing macular carotenoids (lutein and zeaxanthin) e.g. Lutein-Vision - Supplements containing Vitamin C, E, zinc, copper, lutein and zeaxanthin e.g. Macutec Essentials, Macu-Vision Plus - Supplements containing omega 3 fatty acids e.g. Lutein-Vision Advanced - None of the supplements listed above are appropriate for a patient at this stage |
| **3** | Mr Veeson is a 75-year-old man who comes to the pharmacy for assistance with some wound care. He is not a regular customer, but as you assist him, he mentions, that despite having fair vision, he has advanced AMD in both eyes and wanted to obtain some supplements that are likely to help him with his AMD.  **WHAT SUPPLEMENTS, IF ANY, WOULD YOU SUPPLY HIM WITH?**   - Supplements containing Vitamins A, C, E and zinc e.g. Blackmores Macu-Vision - Supplements containing macular carotenoids (lutein and zeaxanthin) e.g. Lutein-Vision - Supplements containing Vitamin C, E, zinc, copper, lutein and zeaxanthin e.g. Macutec Essentials, Macu-Vision Plus - Supplements containing omega 3 fatty acids e.g. Lutein-Vision Advanced - None of the Supplements above are appropriate at this stage |
| **4** | **What dietary advice would you provide to patients who have ESTABLISHED EARLY AMD?**   - Eat a diet rich in leafy green vegetables and have oily fish at least twice a week - Eat a diet low in leafy green vegetables and have oily fish at least twice a week - Eat a diet rich in leafy green vegetables and avoid eating oily fish - Eat a diet low in leafy green vegetables and avoid eating oily fish - None of the above as diet does not influence macular degeneration |
| **5** | **What dietary advice would you provide to patients who are AT RISK OF DEVELOPING AMD?**   - Eat a diet rich in leafy green vegetables and have oily fish at least twice a week - Eat a diet low in leafy green vegetables and have oily fish at least twice a week - Eat a diet rich in leafy green vegetables and avoid eating oily fish - Eat a diet low in leafy green vegetables and avoid eating oily fish - None of the above as diet does not influence macular degeneration |
| **6** | When providing dietary advice, would you consider the patients’ smoking history?   - Yes - No |
| **7** | Which of the following preventive measures has NOT been associated with lowered odds of developing AMD?   - Smoking cessation or not smoking - Healthy diet - Low Body Mass Index - Physical Exercise - Daily 8 hours sleep |
| **8** | **Which of the following is NOT a symptom of AMD?**   - Distorted vision - Dark or empty spaces blocking the central field of vision - Dimming of colour vision - Visual hallucinations - Dark or empty spaces blocking the peripheral field of vision |
| **9** | **Is the statement ‘there are no treatments for DRY AMD’:**   - True - False |
| **10** | **Is the statement ‘there are no treatments for WET AMD’:**   - True - False |
| **11** | Which of the following is not a known risk factor for the development of AMD?   - Parental history of AMD - Having a sibling with AMD - Smoking history - Age 75 years and above - Refractive index errors and wearing glasses |
| **12** | Sudden significant changes in vision are more likely caused by the:   - ‘Dry type’ macular degeneration - ‘Wet type’ macular degeneration |

| SECTION 3: ROLE OF PHARMACISTS IN AMD/VISION IMPAIRMENT/VISION LOSS |
| --- |

| THE FOLLOWING SECTION CONTAINS A LIST OF 12 STATEMENTS. FOR EACH STATEMENT PLEASE SELECT THE OPTION ON THE FOLLOWING SCALE THAT IS MOST APPROPRIATE BY PLACING ‘X’ IN THE COLUMN CORRESPONDING TO THE SCALE MARKER YOU AGREE WITH. | | | | | |
| --- | --- | --- | --- | --- | --- |
| **Statement** | **Strongly disagree** | **Disagree** | **Neutral** | **Agree** | **Strongly**  **agree** |
| 1. Pharmacists should actively screen older patients for risk of vision disorders such as AMD |  |  |  |  |  |
| 1. Pharmacists should spend time demonstrating the use of eye drops in people with vision disorders |  |  |  |  |  |
| 1. Pharmacists need to modify counselling when showing people with vision disorders how to use eye drops |  |  |  |  |  |
| 1. People with vision disorders can be identified when they come into a pharmacy because they are usually using a cane, have guide dogs or wear dark glasses |  |  |  |  |  |
| 1. Pharmacies should explore and invest in assistive technologies that can help those with low vision use medications or adhere to them |  |  |  |  |  |
| 1. Pharmacies should ensure that entry, exits, payment and interface points are accessible for people with vision disorders |  |  |  |  |  |
| 1. Pharmacies should have all staff trained in communication with people who have low vision |  |  |  |  |  |
| 1. People with low vision do not need specialised assistance at pharmacies as they obtain specialised help elsewhere |  |  |  |  |  |
| 1. When counselling people with vision disorders, it is important to speak loudly and use extremely simple language |  |  |  |  |  |
| **Statement** | **Strongly disagree** | **Disagree** | **Neutral** | **Agree** | **Strongly agree** |
| 1. Pharmacists need to enact a key role facilitating self-management and coping skills in people with low vision |  |  |  |  |  |
| 1. When a person with low vision presents at the pharmacy with a carer it is important that pharmacists counsel the carer rather than the patient |  |  |  |  |  |
| 1. People with vision disorders are difficult to deal with in pharmacies |  |  |  |  |  |

| SECTION 4: PERCEPTIONS ABOUT COMMUNICATION WITH INDIVIDUALS WHO HAVE A DISABILITY |
| --- |

| THE FOLLOWING SECTION CONTAINS A LIST OF 20 STATEMENTS. FOR EACH STATEMENT PLEASE SELECT THE OPTION ON THE FOLLOWING SCALE THAT IS MOST APPROPRIATE BY PLACING ‘X’ IN THE COLUMN CORRESPONDING TO THE SCALE MARKER YOU AGREE WITH. | | | | | | |
| --- | --- | --- | --- | --- | --- | --- |
| Statement | **I Disagree Very Much** | **I**  **Disagree Some**  **What** | **I Disagree A Little** | **I Agree A Little** | **I**  **Agree Some**  **What** | **I Agree Very Much** |
| 1. It is rewarding when I can help an individual with a disability. |  |  |  |  |  |  |
| 1. It hurts me when an individual with a disability wants to do something and cannot. |  |  |  |  |  |  |
| 1. I feel frustrated when I do not know how to help an individual with a disability. |  |  |  |  |  |  |
| 1. Contact with an individual with a disability reminds me of my own vulnerability. |  |  |  |  |  |  |
| 1. I wonder how I would feel if I were an individual with the same disability. |  |  |  |  |  |  |
| 1. I feel ignorant about individuals with a disability. |  |  |  |  |  |  |
| 1. I am grateful I do not have a disability. |  |  |  |  |  |  |
| 1. I try to act normally and ignore the disability that the individual has. |  |  |  |  |  |  |
| 1. I feel uncomfortable and find it hard to relax when communicating with an individual with a disability. |  |  |  |  |  |  |
| 1. I am aware of the problems that individuals with a disability have. |  |  |  |  |  |  |
| **Statement** | **I Disagree Very Much** | **I**  **Disagree Some**  **What** | **I Disagree A Little** | **I Agree A Little** | **I**  **Agree Some**  **What** | **I Agree Very Much** |
| 1. I cannot help staring at individuals with a disability. |  |  |  |  |  |  |
| 1. I feel unsure as I do not know how to behave around individuals with a disability. |  |  |  |  |  |  |
| 1. I admire the ability of individuals with a disability to cope. |  |  |  |  |  |  |
| 1. I do not pity individuals with a disability. |  |  |  |  |  |  |
| 1. After frequent contact, I find I just notice the person not the disability. |  |  |  |  |  |  |
| 1. I feel overwhelmed with discomfort about my lack of the disability. |  |  |  |  |  |  |
| 1. I am afraid to look at the individual with a disability straight in the face. |  |  |  |  |  |  |
| 1. I tend to make contacts only brief and finish them as quickly as possible with individuals with a disability. |  |  |  |  |  |  |
| 1. I feel better with individuals with a disability after I have discussed their disability with them. |  |  |  |  |  |  |
| 1. I dread the thought that I could end up like an individual with a disability. |  |  |  |  |  |  |

**Thank you for your valuable participation in this study.**
